# The place of algae in agriculture: policies for algal biomass production

**DOI:** 10.1007/s11120-014-9985-8

**Published:** 2014-03-06

**Authors:** Emily M. Trentacoste, Alice M. Martinez, Tim Zenk

**Affiliations:** 1 Scripps Institution of Oceanography, University of California-San Diego, 9500 Gilman Dr., La Jolla, CA 92093 USA; 2 Sapphire Energy, Inc., 3115 Merryfield Row, San Diego, CA 92121 USA; 3 Algae Biomass Organization, 125 St. Paul Street, Preston, MN 55965 USA

**Keywords:** Algae biomass, Agriculture, Policy, Renewable energy, Algae cultivation

## Abstract

Algae have been used for food and nutraceuticals for thousands of years, and the large-scale cultivation of algae, or algaculture, has existed for over half a century. More recently algae have been identified and developed as renewable fuel sources, and the cultivation of algal biomass for various products is transitioning to commercial-scale systems. It is crucial during this period that institutional frameworks (i.e., policies) support and promote development and commercialization and anticipate and stimulate the evolution of the algal biomass industry as a source of renewable fuels, high value protein and carbohydrates and low-cost drugs. Large-scale cultivation of algae merges the fundamental aspects of traditional agricultural farming and aquaculture. Despite this overlap, algaculture has not yet been afforded a position within agriculture or the benefits associated with it. Various federal and state agricultural support and assistance programs are currently appropriated for crops, but their extension to algal biomass is uncertain. These programs are essential for nascent industries to encourage investment, build infrastructure, disseminate technical experience and information, and create markets. This review describes the potential agricultural policies and programs that could support algal biomass cultivation, and the barriers to the expansion of these programs to algae.

## Introduction

Algae are simple, photosynthetic, generally aquatic organisms that, like plants, use energy from sunlight to sequester carbon dioxide (CO_2_) from the atmosphere into biomass through photosynthesis. Plants evolved from ancient algae ancestors, and the photosynthetic machinery in both plants and algae originally came from the same source: cyanobacteria (Falcón et al. 2010; Fehling et al. 2007). Although algae and plants differ in many ways, the fundamental processes, such as photosynthesis, that make them so distinguished among Earth’s organisms and valuable as crops, are the same.

Certain strains of algae have been used for anthropogenic purposes for thousands of years, including as supplements and nutraceuticals (Kiple and Ornelas 2000) and in the fertilization of rice paddies (Tung and Shen 1985). As early as the 1940s, other strains were identified as possible fuel sources (Borowitzka 2013a) because of their ability to produce fuel or fuel precursor molecules. Large-scale production and cultivation systems, including photobioreactors and outdoor open ponds, were developed in the early 1950s in the U.S., Germany, Japan, and the Netherlands (Borowitzka 2013b; Tamiya 1957). By the onset of the U.S. Department of Energy’s (DOE) aquatic species program (ASP) in the U.S. in 1980, various species of microalgae and cyanobacteria were being produced and farmed on commercial scales around the world, and had been for over 20 years, mostly for the health food and nutritional supplement industries (Borowitzka 2013b).

Microalgae have evolved to be practically ubiquitous throughout the globe, and their varied distributions and evolutionary histories (Fehling et al. 2007) are reflected in extremely diverse metabolic capabilities between species (Andersen 2013). These diverse metabolisms produce a myriad of compounds with anthropogenic relevance including nutraceuticals, such as the carotenoids produced by *Dunaliella* and *Haematococcus* (Borowitzka 2013a, b), the polyunsaturated fatty acids (PUFAs) produced by various species (Ratledge 2004), and the high-value proteins and carbohydrates available in whole-cell supplements of *Spirulina* and *Chlorella* (Görs et al. 2010; Khan et al. 2005). Some microalgae produce compounds of biotechnological interest including fluorescent compounds, such as phycoerythrin, and many produce isoprenoid molecules that can be used in food and over-the-counter products (Andersen 2013).

Microalgae have also been identified as attractive sources of biofuel because different species can produce a variety of fuel products. Various microalgal species have the ability to produce large quantities of lipid while sequestering CO_2_, particularly neutral lipids in the form of triacylglycerol (TAG), which can be converted to fatty acid methyl esters (FAMEs), the main components of biodiesel (Hossain et al. 2008), through trans-esterification, or refined into other fuel constituents (Pienkos and Darzins 2009). Total lipids and other biomass constituents can be converted into crude oil alternatives through thermochemical processes such as hydrothermal liquefaction (Barreiro et al. 2013). Microalgal carbohydrates can be fermented into ethanol, and some species can produce biohydrogen (Radakovits et al. 2010). In addition to their diversity of products, microalgae are attractive as fuel sources because many species grow relatively fast compared to terrestrial plants and can be grown on brackish or saline water, thus avoiding the use of unsustainable quantities of freshwater, an increasingly limited resource (Dismukes et al. 2008). Table 1 provides an overview of some commercial algal products and potential sources.

**Table 1:** Commercial products from algae

Product	Use	Example source	Reference
β-Carotene	Supplement	*Dunaliella*	Lamers et al. (2008)
Astaxanthin	Supplement	*Haematococcus*	Lorenz and Cysewski (2000)
Whole-cell nutraceuticals	Supplement	*Spirulina*	Khan et al. (2005)
		*Chlorella*	Görs et al. (2010)
Aquaculture feed	Animal feed	*Tetraselmis*	Gladue and Maxey (1994)
		*Isochrysis*	Gladue and Maxey (1994)
Polyunsaturated fatty	Supplement	*Crypthecodinium*	Jiang et al. (1999)
acids (PUFAs)		*Shizochytrium*	Spolaore et al. (2006)
Phycoerythrin	Biotechnology	Red algae	Pulz and Gross (2004)
Fuel molecules	Energy	*Botryococcus*	Ashokkumar and Rengasamy (2012)
		*Scenedesmus*	Mandal and Mallick (2009)
		*Neochloris*	Gouveia et al. (2009)
Anticancer drugs	Pharmacueticals	*Symploca*	Coates et al. (2013)

**Fig. 1:**
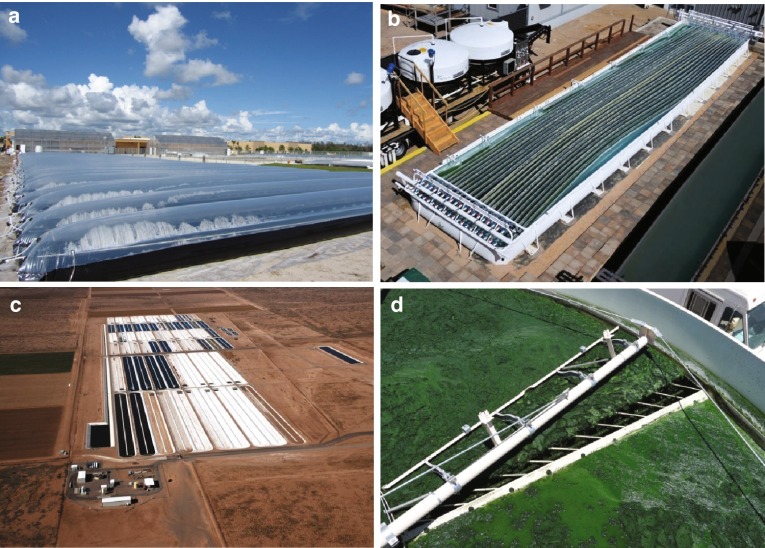
Algaculture in the U.S. Algaculture can take place in closed photobioreactors, like those of Algenol in Florida (**a**) and Solix Biosystems in Colorado (**b**), or in open ponds like those of Sapphire Energy, Inc. in New Mexico (**c**). Like agriculture, algae cultivation requires growth as well as harvesting infrastructure, such as that of Sapphire Energy Inc. (**d**)

Algaculture, or the farming of algae (Savage 2011), merges the requirements of traditional terrestrial plant agriculture such as sunlight, water, CO_2_, nutrient inputs, and harvesting systems with additional aquaculture requirements such as self-contained aquatic systems, water quality, and waste disposal/recycling (Fig. 1). Because of their capability to produce commodities that span multiple markets, including those of health food, nutraceuticals, pharmaceuticals, animal feed, chemicals and energy, algae are uniquely versatile crops (Rosenberg et al. 2008). These diverse metabolic capabilities are due, in part, to the diversity of strains found within the algal lineage. Algae strains grown for food purposes, such as *Spirulina*, have a starkly different metabolic profile from strains grown for energy, such as *Scenedesmus*. The diversity of their end products, and their cultivation using both agriculture and aquaculture practices make algae unique among other agricultural products.

Despite significant overlap with both traditional agriculture and aquaculture (which Congress has defined as agriculture, including that of aquatic plants) (Food and Agriculture Act of 1977, 1977), algaculture has not yet been afforded an official position within Title 7 of the U.S. Code (USC) for Agriculture. There are currently a number of other crops that share commonalities with algae in their cultivation practices or diversity of end-use markets, but these have all been designated a place within Title 7. For example, the commercial cultivation of aquatic plants, such as seagrass, is eligible for a diverse array of agricultural programs. Similarly, the farming of terrestrial crops for renewable energy, which shares the same end market and purpose as many algal-farming operations, benefits from its definition as agriculture.

**Fig. 2:**
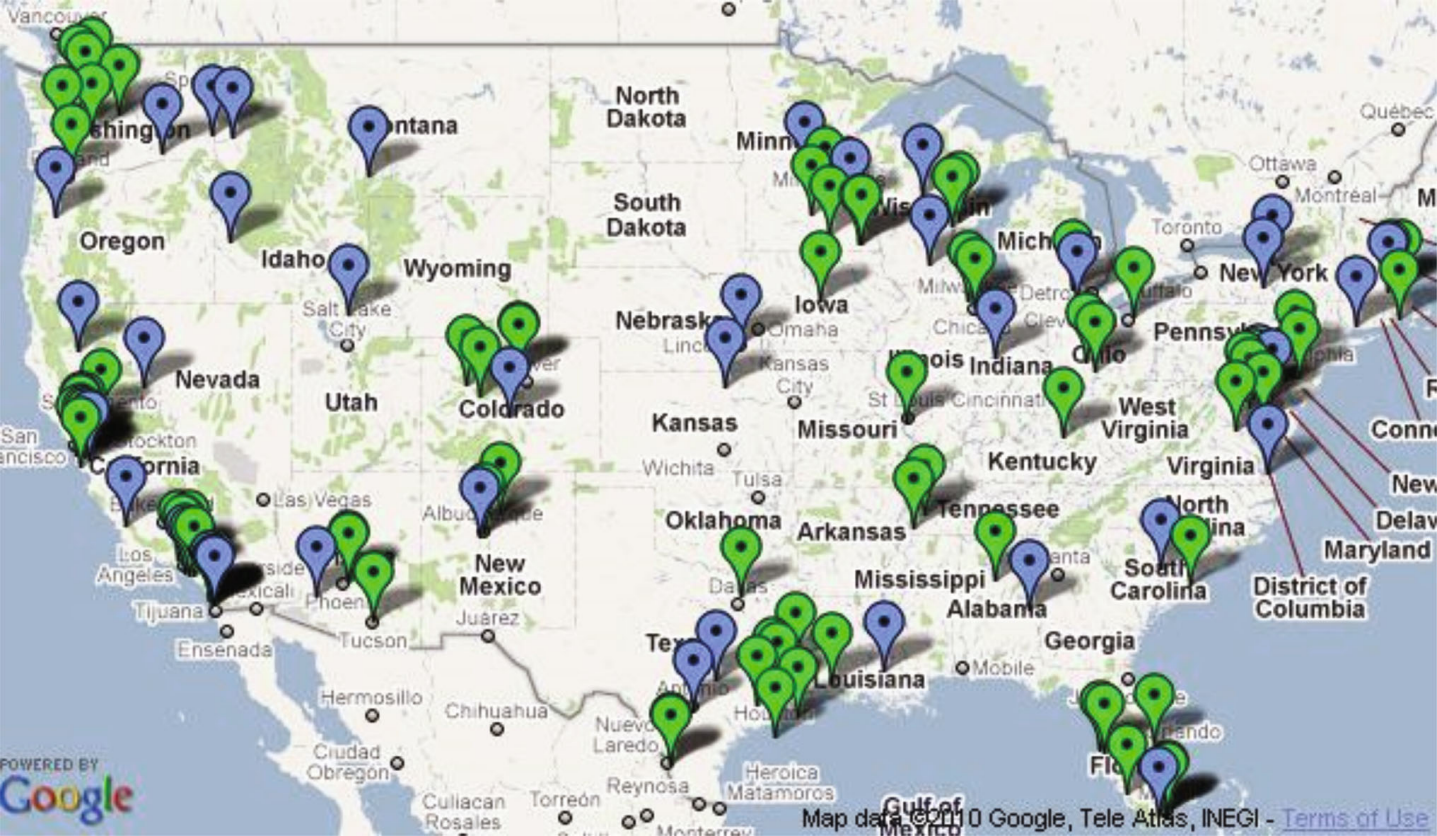
Algae projects in the U.S. Algal biomass projects exist in almost every state in the U.S. *Blue pins* denote a research institution, *green* denote a private project or company

Funding for research and development of algal biomass cultivation has increased over the last decade, and has led to the emergence of research programs, private projects, demonstration- and commercial-scale facilities across the U.S. (Fig. 2). The increase is primarily due to the growth of the algal biofuel industry in response to the demand for alternative fuel sources driven by the renewable fuel standards (RFS) (Tyner 2013). While the use of algae as functional food or feed ingredients is also on the rise (Ibañez and Cifuentes 2013), there are currently few federal program resources focused in this area. The production of algae for any end product is a two-phase process involving the farming and cultivation of algal biomass followed by processing of the harvested biomass. The ability of the algal biomass industry to access federal programs that support the agricultural phase is imperative for future growth. This report analyzes the place of algae in the current agricultural policy and funding landscape, and the opportunities and pitfalls that exist for algae within this policy framework.

## Agricultural programs

Congress has legislated a number of renewable energy programs that can be applied to algae such as the Bioenergy Program for Advanced Biofuels, the Rural Energy for America Program, the Biomass Research and Development Initiative and various grants and loans established in the 2008 Farm Bill in section 9003 of the USC (Food, Conservation, & Energy Act of 2008, 2008). These programs, however, focus on research and development of algae for fuels at smaller scales. While this initial investment in research & development (R&D) is essential to build knowledge, expertise, and technology around algae, the industry is now entering the formative stage of large-scale commercialization, which requires broader coordination among federal agencies and support infrastructure to gain proper alignment at the federal and state level required for a successful industry.

### Biomass crop assistance program

The Biomass Crop Assistance Program (BCAP) was established in the 2008 farm bill (Food & Conservation Act of 2008, 2008) to financially assist farmers wishing to establish, produce, and deliver biomass feedstocks. BCAP’s purpose is to promote farming of bioenergy crops. The program provides either one-time establishment payments, annual payments, or matching payments to help with harvest, storage, and transportation of biomass. Proposals for BCAP funding are submitted to the FSA and can come from either producers or conversion facilities (Schnepf 2011). While many traditional biofuel crops are currently eligible for BCAP funding, such as switchgrass and most non-food biomass, the 2008 farm bill specifically excluded algae from participation in the matching payment side of BCAP but qualifies algae for establishment payments through BCAP (Food & Conservation Act of 2008, 2008).

### Support programs

Congress has appropriated numerous federal agencies, such as the USDA and DOE, funds and authorization to implement programs that aid and support development of agriculture and aquaculture resources (Table 2). Since the passage of the original Agricultural Adjustment Act of 1933, each subsequent farm bill has evolved to address rising relevant issues in agriculture. This frequently involves drafting new programs or expanding existing programs to the new developing technologies. The 1977 farm bill (Food & Agriculture Act of 1977, 1977) expanded the definition of agriculture to include aquaculture, thus spurring the development of industry in the U.S. The 2002 farm bill was the first to include a title (9003) on energy (Farm Security & Rural Investment Act of 2002, 2002), enabling the initial research and development of biofuels and bioenergy and set the stage for bio-based energy standards in the 2005 and 2007 energy bills.

**Table 2:** Overview of federal support programs

Agricultural and energy support program provided by the Farm Service, USDA and DOE. Shaded circles signify all feedstocks within that crop group are eligible for a particular service, empty circles signify no feedstocks within that crop group are eligible, and half-shaded circles signify only certain feedstocks within that crop group are eligible. For example, farm service programs are only available for algal biomass feedstocks that are used to produce food or feed commodities

The current farm bill, primarily through the arm of the USDA and associated agencies, funds a large number of assistance programs for agriculture and aquaculture (Agricultural Act of 2014, 2014). All of the major farm price and income support programs comprising the farm safety net are available only to the “program crops” of corn, cotton, wheat, tobacco, peanuts, rice, and some new oil crops such as sunflower and oilseed. The main farm safety net programs restricted to program crops include the Marketing Assistance Loan, Price Loss Coverage, and Agriculture Risk Coverage. Additional programs, such as the Feedstock Flexibility Program for sugar, also instill price control while simultaneously attempting to bridge the gap with biofuel producers looking to meet RFS standards. These programs ensure that market prices for program crops never fall below a certain limit and provide direct income support or revenue assistance. Farmers of specialty crops, such as fruits and vegetables, aquaculture crops, horticulture crops, and livestock are eligible for a range of support programs outside of the safety net. These programs provide extension services, loans, crop insurance, and incentives for improving environmental quality of farms (Mercier 2011).

### Extension services

Some of the most important benefits allotted to agriculture and aquaculture in the U.S. are research, teaching, and extension services. Extension services are some of the oldest programs in U.S. agriculture, dating back to the Smith-Lever Act of 1914 that established a link between universities and the USDA (Smith-Lever Act 1914). The purpose of the programs has always been to (1) develop applications for agricultural research and (2) provide instruction on agricultural technologies to farmers. Today, the Cooperative Extension Service program of the USDA provides funding through the National Institute of Food and Agriculture to support programs that connect scientific agricultural research with local farmers. Extension services are administered through regional offices that bring expertise from land-grant universities to local levels to instruct farmers in emerging technologies that can increase productivity.

Extension services are essential for disseminating information about innovative research and technologies throughout the agricultural industry. They also play an extremely important role in providing more immediate assistance to issues faced by local farmers and in developing plans that address regional problems. The application of USDA’s extension services to aquaculture in the 1981 farm bill was instrumental in expanding the industry and coordinating research and commercialization efforts (Agriculture and Food Act of 1981).

### Federal crop insurance programs

The additional support programs available for all farmers are important for the continuing success of non-program crops. These programs provide assistance for the development, commercialization, and continuation of farms and provide incentives for environmentally sound farming practices. The largest of these programs, in which all farmers (including those of aquaculture and livestock) can participate, is the crop insurance program. The original crop insurance program began in 1938 and only covered major crops (Agricultural Adjustment Act of 1938, 1938), but the passing of the Federal Crop Insurance Act of 1980 expanded the program to be universal (Federal Crop Insurance Act of 1980, 1980). Crop insurance is run by the USDA Risk Management Agency (RMA) and paid for by the separate Federal Crop Insurance Corporation (FCIC).

Over 100 crops are currently eligible for the Federal Crop Insurance (FCI) program, in which farmers pay a subsidized premium for insurance delivered by private companies. While program crops are eligible for revenue-based loss insurance, specialty crops typically only participate in physical crop-loss insurance. If a crop is ineligible for the program, then it can still be insured through the Non-insured Crop Disasters Assistance program, established in the 1996 farm bill and run by the Farm Service Agency (FSA), which functions similarly to FCI (Federal Agriculture Improvement & Reform Act of 1996, 1996). Sea grass, a similar crop to algae that requires a blend of agriculture and aquaculture, is eligible for Non-Insured Crop Disasters Assistance (FSA 2011). Additional insurance support is available for all farmers to cover losses from natural disasters under the Supplemental Revenue Assurance Program. This program provides additional assistance beyond crop insurance to farmers who experience a decrease in revenue due to natural disasters and is only available for crops that are enrolled in one of the crop insurance programs.

The expansion of crop insurance programs to specialty crops, aquaculture, and livestock was important for the development and protection of these industries. Farms of these commodities are all affected by the same environmental factors as those of program crops, such as lower-than-expected production due to droughts, natural disasters, soil quality, water availability, etc. The farming of algae is equally susceptible to different but similar factors that affect biomass and crop yields.

### Farm loan programs

Farm loans are essential in successful agriculture as up-front capital is needed to make purchases of inputs such as fertilizer, equipment, land, etc. Most farm loans are authorized by the Consolidated Farm and Rural Development Act (1961) and can be in the form of direct loans, guaranteed loans or emergency loans. Direct loans cover input purchases and farmland purchases, require farmers to complete financial training courses and are given preferentially to beginning farmers. Guaranteed loans are available in coordination with banks and emergency loans can help cover natural disasters.

### Environment and conservation programs

Agriculture, aquaculture, and livestock farms have traditionally been eligible for a number of federal programs that incentive environmentally friendly practices and resource conservation. Most notable, the Environmental Quality Incentives Program (EQIP), introduced in the 1996 farm bill, provides technical and financial assistance to farmers to increase the environmental quality of their farmland. EQIP funds are distributed by states in competitive programs that focus either on innovation of novel conservation practices or water enhancement, including enhancing water quality and conservation. EQIP also works in partnership with farms to aid in farm design that promotes environmental quality and resource conservation.

The Conservation Stewardship Program (CSP) awards funds to farmers that have adopted uncompensated practices across their entire operation for overall conservation. To be eligible for CSP funds, farmers must be sustaining conservation of a certain resource and must demonstrate improvement and maintenance of conservation practices. Farmers can receive both EQIP support and CSP rewards. The final environmental program, the Agricultural Management Assistance (AMA) Program was established in the Agricultural Risk Protection Act of 2000 to address the fact that crop insurance is heavily concentrated among program crops in only a few states. The AMA provides assistance for conservation practices in a select 16 states.

The algae industry, which has most recently been associated with renewable energy production with the added constraints of reducing greenhouse gas emissions and being cost-competitive with fossil fuels, has already made substantial technological advances in freshwater conservation and nutrient recycling for commercial-scale production. In order to be categorized as advanced biofuel, the overall process of algal fuel production must represent a 50 % decrease in GHG emission compared to fossil fuels (Energy Independence & Security Act of 2007, 2007). A study conducted by the University of Virginia found that commercial scale production of algae-to-energy can result in a 68 % reduction in overall greenhouse gas emissions when compared to traditional fossil petroleum (Liu et al. 2013). Additionally, to increase economic feasibility, algae can be grown on non-potable saline or wastewater and nutrients can be recycled, drastically mitigating freshwater use and fertilizer inputs. The company BioProcess Algae, for example, has successfully utilized waste outputs of water, heat, and CO_2_ from corn ethanol fermentation to cultivate algal biomass for various end products. Coupling algae cultivation with waste outputs from other industrial processes provides cost-effective and sustainable solutions to cultivation barriers.

### Marketing services

Agricultural products are frequently subjected to market analyses by the USDA such as economic and census reports. As the commercialization of algae progresses, market analyses will be advantageous to assess the strengths and weaknesses of the industry, the interplay between the agricultural and energy aspects of algae, and the outlook of the industry. The USDA also provides marketing assistance to farmers through financial assistance, research and promotion (AMS 2013). To successfully break into the agricultural market, algae would benefit from the marketing services available from the USDA.

### State programs

Defining the commercial cultivation of algae as agriculture provides opportunities at the state level as well. Many states offer additional loan and financing programs, especially for first-time farmers, such as “Aggie Bonds” that encourage private lenders to loan to beginning farmers (CDFA 2005). Beyond financial assistance, states can control laws associated with agricultural property and zoning. For example, the Ohio state legislatures recently defined algaculture as agriculture to allow use value assessments of algae cultivation land for tax purposes, thus lowering property taxes for land used for commercial algaculture (OH-H.R. 2012). The law additionally limits the authority of zoning laws to restrict algae cultivation on lands. Although decisions on specific investments in algae development are made at the regional and local levels, a federal initiative is still imperative to establish and influence direction and focus for the industry, as well as to develop guidance for new algae programs.

## Application of agricultural programs to algae

Opportunities currently exist for algae cultivation to expand commercialization within agriculture if it were defined as such. The most notable is the potential to fill a large void in agriculture of the use of non-arable land to produce renewable hydrocarbons and high value protein. Unlike terrestrial crops, algae do not require fertile soil or arable land for growth, thus expanding the areas of the country in which algae can be cultivated. Algae do require other inputs such as salt or freshwater, nutrients, and consistent year-round sunlight. Taking all of these factors into account, a recent study by the Pacific Northwest National Laboratory (PNNL) identified ~90,000 sites in the U.S. that would be suitable for algaculture, comprising ~5.5 % of the contiguous U.S. land mass and consisting predominantly of shrub/scrub landscape. These sites exclude any cropland, urban land, protected lands, wetlands, wilderness, or significantly sloping landscapes (Wigmosta et al. 2011). To compare, agricultural land currently utilizes over 40 % of the total U.S. land mass.

**Fig. 3:**
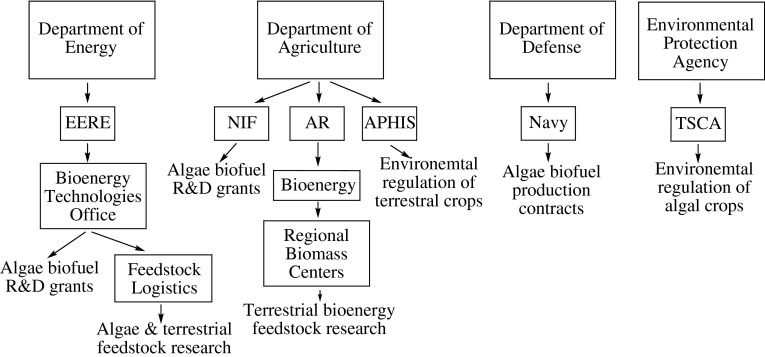
Federal agency jurisdiction over algae versus terrestrial crops. Four different federal departments hold jurisdiction over various aspects of algae cultivation, research, and products. *EERE* energy efficiency & renewable energy, *NIFA* National Institute of Food & Agriculture, *ARS* Agricultural Research Service, *APHIS* Animal & Plant Health Inspection Service, *TSCA* toxic substance control act

The USDA currently asserts jurisdiction of algae as an agricultural crop, and can potentially offer agricultural safety net programs to algal biomass companies. Despite the role of the USDA in overseeing agricultural programs for algae, barriers still exist to the application of these programs. Many of these barriers exist at the federal and state levels, and stem from lack of an overall national plan for the development of algaculture, from the overlapping jurisdictions of other federal agencies over different aspects of algae cultivation, (Fig. 3), and from the diverse end products generated by algae.

Agencies that currently hold some responsibility over algae are the DOE, USDA, DOD, and EPA. The DOE has been involved in algae biofuel research since the onset of the 25-year long ASP in 1980 and has done extensive research on both algal biology and large-scale cultivation under its Biomass Program (Sheehan et al. 1998). Findings have been reported in both the ASP close-out report and the National Algal Biofuels Technology Roadmap (U.S. DOE 2010). The DOE also appropriates funding for grants and loans to industry and academic partners doing algae biofuel R&D. The DOD appropriates R&D grants and participates in demonstrations for algal biofuel use. It has currently entered contracts for developing commercial-scale production. While the USDA is responsible for regulatory oversight and approval, biotechnology and environmental regulation of genetically modified crops, the EPA has asserted jurisdiction for the permitting of genetically engineered algae varieties under its Toxic Substance Control Act, further supporting the notion of uncoordinated and overlapping federal support and regulation of the algae industry. There are also statutory limitations for the USDA’s support of algae. Existing law, although not defined well and left open to individual programs for interpretation, may have the ability to support algae when used to produce a feed or food; the same standard, however, is not applied to algae if the end product is used to produce energy. None of these inconsistencies exist for the program crops (e.g., corn); they qualify for the vast array of USDA assistance no matter what products they support.

The USDA asserts responsibilities for agricultural policies pertaining to algae, but the end-use of algae as an energy source has created uncertainty in the applicability of these policies to algae cultivation. While a clear case can be made for expanding these programs for algal biomass used for food and nutraceutical purposes, there are still holes in the existing framework to accommodate algal biomass grown for bioenergy purposes. Because algae are such unique crops in their diversity of end product potential, no precedent exists to determine if a particular algae cultivation facility is eligible for agricultural programs or not. The USDA currently has no clear methodology for evaluating algal biomass producers within the agricultural landscape.

The uncertainty in algae’s eligibility under agriculture is further exacerbated by insufficient communication about algal policies between the USDA’s national leadership and its state and regional offices. The USDA’s work, including decisions on application of policies to various USDA state offices, is primarily carried out in the field through more local offices, but while the national office claims jurisdiction over algae, there is again no precedent for state offices to follow. For example, the USDA’s five Regional Biomass Centers, which are designed to lead research in sustainable biomass production, currently specifically exclude algae to avoid DOE overlap (Steiner 2011). Extension services, such as those provided under the Smith-Lever Act, would be appropriate to link regional USDA centers with local institutions and algae cultivators to develop methodology for evaluating algal biomass production under the agricultural framework.

Another notable barrier is the lack of an overall algae-specific plan to move algae past R&D and into the formative stages of commercialization. The DOE has written an algae-specific roadmap, but this is primarily a summary of technologies that were available at the time and directions for R&D, without specific suggestions for moving into development and commercial stages (U.S. DOE 2010). Since then, a number of reports have been published agreeing that commercialization of algae, particularly for biofuels, is feasible given certain improvements in the production process (NRC 2012; ANL et al. 2012). Furthermore, since these reports, many of these improvements have been made and technologies have been developed that successfully demonstrate the ability to sustainably cultivate and harvest algae on large scales. While continued R&D is imperative to maintain and drive such improvements in the overall production process, it is now more important than ever for federal agencies to map out the next stage of the scale-up process.

The overlapping jurisdiction of algae, lack of a national plan, and specifically the assumption of major responsibility by the DOE, has caused the focus of algal policies to primarily revolve around its downstream use for energy, and to overlook expansion of policies that would support its most basic properties as a crop. Consistent, long-term federal policies are essential for scaling up biomass production of algae for energy, carbohydrates, protein and many other products (U.S. DOE 2012). The farming of algae requires biology, cultivation, harvest, and biomass processing practices, modeled after agricultural systems, which require independent and unique support networks for commercialization from those required for the downstream conversion of biomass into fuel (such as extraction, conversion, and biorefining processes).

### Looking forward

While we have discussed the successes for algae in the U.S. agricultural framework and the pitfalls that still exist, we can also identify areas of progress. Individual states have taken initiative to pave the way in recognizing algae cultivation as agriculture. In 2012 two states, Arizona and Ohio, specifically amended their laws to define algaculture as part of agriculture. While these changes had different specific effects in each state, they were both carried out with the purpose of increasing investment in algaculture and attracting the industry to those states. In Ohio, the recognition of algae farming as agriculture allows land used for algae cultivation to be eligible for the same land use valuation as agriculture, thus allowing lower property taxes for algae farms. It also limits the authority of zoning laws to restrict algaculture on lands. The Ohio legislation was proposed with widespread support from many factions including the Farm Bureau, the Poultry Association and the Soybean Association (OH-H.R. 2012). In Arizona, state trust lands can now be leased for algaculture, and algae farmland is eligible for lower property taxes afforded to traditional farmland (AZ-HR 2012a, b). In 2013, Iowa also passed a similar bill defining land used for algal cultivation as agricultural (IA-H.R. 2013).

Arizona’s bills have allowed for the development of a national test bed for algal biomass production, led by Arizona State University. This multi-regional private and public partnership, funded by the DOE, focuses on developing algae cultivation on large, economically relevant scales and involves coordination between facilities in Arizona, Ohio, California, Hawaii, and Georgia. Other public–private partnerships include the California Center for Algal Biotechnology, which coordinates and promotes research, commercialization and public education projects.

## Conclusions

Large-scale cultivation of algae, or algaculture, has existed for over half a century. More recently, algaculture for food and fuel purposes has begun the transition from R&D and pilot-scale operations to commercial-scale systems. It is crucial during this period that institutional frameworks (i.e., policies) support and promote development, and commercialization. While the U.S. government has supported the R&D stage of algaculture for biofuels over the last few decades, it is imperative that policies anticipate and stimulate the evolution of the industry to the next level.

**Fig. 4:**
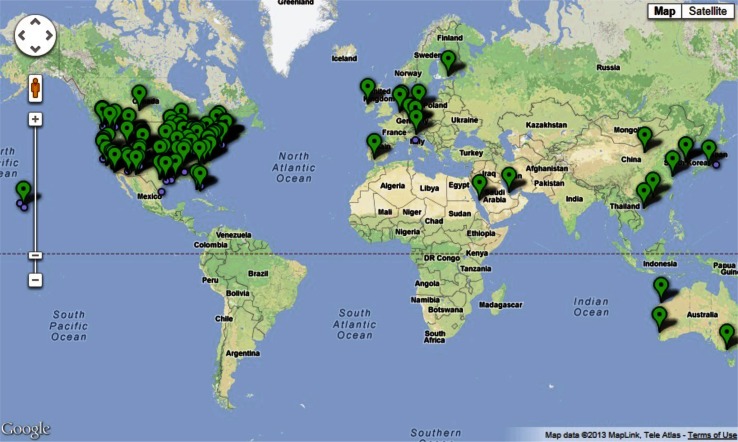
The global algal biomass industry. Locations of algal biomass projects, production, and companies around the world

Large-scale cultivation of algae merges the fundamental aspects of traditional agriculture and aquaculture. Despite this overlap, algaculture has not yet been afforded an official position within agriculture or the benefits associated with it. Recognition of algaculture as part of agriculture under the USDA at national, regional, and local levels will expand agricultural support and assistance programs to algae cultivation, thus encouraging progression of the industry. The U.S. is currently the world leader in algal biomass technology and hosts a disproportionate number of companies devoted to the industry (Fig. 4). Continued federal support and initiatives will provide the spark needed to drive algaculture into the next stage of commercialization.
